# Retrograde Intramedullary Nailing with a Blocking Pin Technique for Reduction of Periprosthetic Supracondylar Femoral Fracture after Total Knee Arthroplasty: Technical Note with a Compatibility Chart of the Nail to Femoral Component

**DOI:** 10.1155/2014/856853

**Published:** 2014-12-11

**Authors:** Ichiro Tonogai, Daisuke Hamada, Tomohiro Goto, Tomoya Takasago, Takahiko Tsutsui, Naoto Suzue, Tetsuya Matsuura, Koichi Sairyo

**Affiliations:** Department of Orthopedics, Institute of Health Biosciences, University of Tokushima Graduate School, 3-18-15 Kuramoto, Tokushima 770-8503, Japan

## Abstract

Periprosthetic fractures after total knee arthroplasty (TKA) present a clear management challenge, and retrograde intramedullary nails have recently gained widespread acceptance in treatment of these fractures. In two cases, we found a blocking screw technique, first reported by Krettek et al., was useful in the reduction of the fractures. Both patients attained preinjury mobility after intramedullary nailing. Moreover, we present a chart summarizing the notch designs of various femoral components because some prosthetic knee designs are not amenable to retrograde nailing. We hope this will be helpful in determining indications for retrograde nailing in periprosthetic fractures after TKA.

## 1. Introduction

The number of periprosthetic supracondylar femoral fractures is increasing due to more total knee arthroplasties (TKAs) being performed on increasingly elderly patients, and postoperative activity and survival are also increasing. Other risk factors for these fractures include steroid use, rheumatoid arthritis, neurological disorders [1], and possibly anterior notching during surgery [2]. The incidence of these fractures ranges from 0.3% to 2.5% [3], and some problems associated with them include a short distal segment for fixation, osteoporotic bone, nonunion, malunion, and malalignment, all of which make treatment challenging [4].

Rorabeck's classification of periprosthetic supracondylar fractures is commonly used to evaluate fracture displacement and prosthetic stability [4]. If there is no evidence of TKA loosening, the main treatment option for supracondylar femoral fractures is plating [5–9] or intramedullary nailing [10–13]. The conventional open plating technique results in vascular disruption, which increases the risk of malunion and mechanical failure [14]. Furthermore, the failure rate of plating is twice that of intramedullary nail fixation [15]. However, intramedullary nailing for these fractures can be technically difficult; obtaining a satisfactory reduction is especially challenging.

To achieve anatomical reduction of supracondylar fractures, in 1999 Krettek et al. [16, 17] reported the Poller (blocking) screw technique as an important adjunct for intramedullary nailing. This technique has subsequently been shown to be effective in aiding fracture reduction [18, 19], but there have been no reports of this technique being applied specifically for the reduction of periprosthetic supracondylar fracture. Here we report 2 cases of such fracture treated with intramedullary nailing and the blocking screw technique that achieved excellent reduction.

Although most modern knee prostheses allow insertion of a retrograde supracondylar nail, it is important to evaluate whether the femoral component can allow it [20]. Therefore, we also reviewed the notch designs of various femoral components and summarize the compatibility of the supracondylar nails and femoral components that can be used.

## 2. Case Report

**Figure 1:**
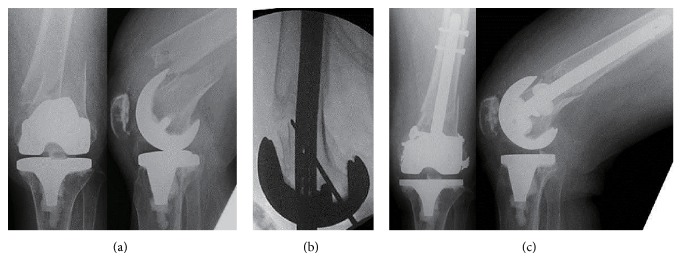
(a) Radiographs of the left displaced periprosthetic supracondylar fracture. (b) A 2.4 mm block pin inserted on the lateral side of the distal fragment. (c) The nail inserted with excellent repositioning of the fracture site.

*Case 1.* A 58-year-old woman with rheumatoid arthritis underwent TKA (Omnifit 3000; Stryker, Kalamazoo, MI) 18 years before presentation. The intercondylar width of the femoral component was 19.5 mm, but the distance from the anterior femoral osteotomy phase to the open box anterior edge of the component was not noted. She presented to us at age 76 with a displaced supracondylar fracture of the left femur, just proximal to the implant (Rorabeck type II) after tripping and falling (Figure 1(a)). We, arthroplasty surgeons, performed fixation using a retrograde femoral nail. A 5 cm vertical skin incision was made just medial to the patellar tendon, and the soft tissues were spread. The entry for the nail was made using a guide wire. Initially, we could not achieve satisfactory reduction with the distal fragment extended in sagittal plane when the nail was inserted between the condyles of the femoral prosthesis. Next, a K-wire of 2.4 mm diameter × 300 mm length was inserted on the lateral side of the distal fragment as a blocking pin to prevent extension deformity and to guide the nail to the center of the distal fragment (Figure 1(b)). Then, a 13 mm diameter × 170 mm length T2 supracondylar femoral nail (Stryker) was inserted with a satisfactory reduction (Figure 1(c)). After the nail was locked, the blocking pin was removed and no cross lock screw was used after fixation. Although recurrent pyogenic spondylitis delayed rehabilitation, the supracondylar femur fracture healed, with *α* and *γ* angles, which indicate the coronal and sagittal alignments of the femoral component on the postoperative radiographs, of 97° and 0°, respectively. At the final followup 2 years after surgery for fracture, she had regained her preinjury mobility.

**Figure 2:**
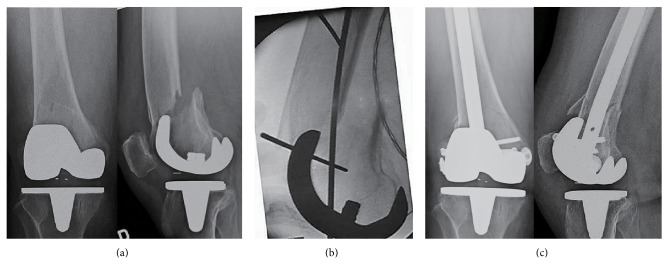
(a) Radiographs of a fracture located proximal to the right femoral component of TKA. (b) On the lateral side, 2.4 mm blocking pins inserted into the distal and proximal fragments. (c) Acceptable reduction and fixation of the fracture with a nail.

*Case 2.* A 71-year-old woman underwent right TKA for treatment of osteoarthritis (LCS; Depuy, Warsaw, IN) 6 years before presentation. The intercondylar width of this femoral component was 17.5 mm, and the distance from the anterior femoral osteotomy phase to the anterior edge of the open box component was 16.3 mm. She presented to our team, which was the same surgical team including the same supervising senior surgeon as with Case 1, at age 77 with a fracture above the prosthesis (Rorabeck type II) after a minor fall (Figure 2(a)). The patient underwent retrograde femoral nail placement with a 13 mm diameter × 200 mm length T2 supracondylar femoral nail (Stryker). Initially the nail did not provide a satisfactory reposition; we inserted two K-wires of 2.4 mm diameter × 300 mm length into the distal and proximal fragments from lateral to medial to prevent a sagittal plane deformity (Figure 2(b)). The fracture site was repositioned well without gap and extension deformity, and the blocking pin was removed after the nail was locked (Figure 2(c)). No cross lock screw was added after fixation. Her recovery was uneventful. The right supracondylar femoral fracture healed, with *α* and *γ* angles of 98° and 3°, respectively. At the final followup 2 years after surgery for fracture, she was able to walk without any aids on a stable knee with a range of movement of 0–90°.

## 3. Discussion

Managing periprosthetic supracondylar fractures is complicated by several factors, including osteoporotic bone in the distal femoral metaphyseal region, distal segments that are short for adequate fixation, nonunion, malunion, and malalignment [4]. A retrograde femoral intramedullary nail is the ideal choice because it provides adequate stabilization of the periprosthetic supracondylar fracture, with minimal disturbance of the fracture hematoma and minimal soft tissue stripping. However, anatomical reduction may not always be possible with standard closed intramedullary nailing because the distal medulla of the femur is wider than the middle diaphysis, and the intramedullary nail cannot completely fill the intramedullary canal [18, 21, 22].

Blocking screws are designed to guide the intramedullary nail to the desired direction by narrowing the intramedullary canal and obtaining better reduction [18, 19]. In our cases, we inserted a 2.4 mm K-wire into the fragment from lateral to medial to prevent a hyperextension deformity and guided the intramedullary nail using the Poller screw technique. This procedure resulted in excellent fracture reduction. Furthermore, the fracture healed without displacement, even though the blocking pins were removed after fixation. To our knowledge, this is the first report demonstrating the efficacy of the blocking pin technique for correcting deformities encountered during intramedullary nailing of periprosthetic supracondylar fractures.

**Figure 3:**
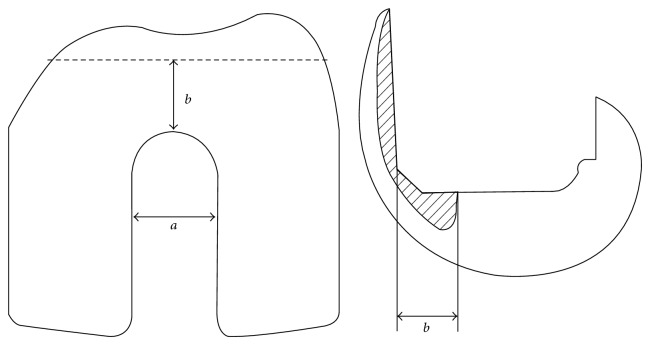
Intercondylar width of the femoral component (a) and distance from the anterior femoral osteotomy phase to the anterior edge of the open box component (b).

Intramedullary devices are not applicable to all designs of TKA. Retrograde intramedullary nail techniques generally require an open box femoral prosthesis.  Figure 3 and Table 1 give a summary of the notch designs of various femoral components, including intercondylar width and distance from the anterior femoral osteotomy phase to the anterior edge of the open box component. As Currall et al. and Heckler et al. reported, we believe that the intercondylar width is one of the most important factors when determining nail compatibility [23, 24]. Because the smallest distal diameter of retrograde nails is 11.5 mm for the T2 supracondylar nail (Stryker) and the TRIGEN short knee nail (Smith & Nephew, Memphis, TN), the intercondylar distance must be more than 12 mm to accommodate the nail [12]. Moreover, the distance from the anterior femoral osteotomy phase to the anterior edge of the open box of the component is also of major importance. If it is long, a deformity may occur in extension of the femoral prosthetic component produced by an overly posterior nail entry point in the intercondylar notch. On the other hand, we also have to be aware that the inserted postcum in the posterior stabilized type may interfere with the nail end, and the peg of the femoral implant may interfere with the most distal locking screw [25]. Note that the compatibility chart shown in Table 1 may not be applicable if the femoral component is placed in an incorrect position or direction.

It is important to plan whether the femoral component will accommodate the nail before surgery. We hope that the chart provided can help plan treatment for periprosthetic supracondylar fractures by providing a quick, easy, and reliable reference of prostheses commonly used for surgeons managing periprosthetic supracondylar femoral fractures when the femoral component is placed in a correct position or direction.

## Figures and Tables

**Table 1:** Intercondylar notch designs of the inserted TKAs and their compatibility with supracondylar nails. All these dimensions were officially provided by the manufactures.

Manufacture & model name of the component	Size of the component	Intercondylar width of the component (mm)	Distance from the anterior femoral osteotomy phase to the open box anterior edge of the component (mm)
Senko Medical (Tokyo, Japan)			
Quest CR	1–5	15–18.6	18.9–24.7
Smith & Nephew (Memphis, TN)			
Genesis II CR	1–8	16.5–19	16–19.9
Genesis II PS	1–6	16.5	11–23
Legion CR	1–8	16.5–19	16–19.9
Legion PS	1–6	16.5	9.5–17
Profix CR	All	20	N/A
Profix PS	All	15	N/A
Zimmer (Warsaw, IN)			
Nexgen CR-Flex	A–G	11.9–13.3	18.9–27
Nexgen LPS	A–G	13.7–21.2	15.3–20
Nexgen LPS-Flex	A–G	13.7–21.2	15.3–20
MG I CR	S–large plus	11–14	N/A
MG II CR	All	12	N/A
IB I PS	All	16	N/A
IB II PS	54–64	15–18	N/A
Stryker (Kalamazoo, MI)			
Scorpio CR, PS	3–13	16.5–20.5	8–17.1
NRG CR	3–13	18–22.2	15.8–26.8
NRG PS	3–13	18–22.2	5.9–17.1
Kinemax Plus CR, PS	X small–large	17–21	9.9–21.6
Omnifit 3000 CR, PS	3–11	19.5	N/A
Delta Fit 7000 CR, PS	3–13	19.5	17.9–20.8
Triathlon CR	1–7	16.1	10–16.5
Triathlon PS	1–7	20.8	10–16.5
Depuy (Warsaw, IN)			
Sigma CR	1.5–5	17.8	12–16.7
LCS RP CR	Small–large plus	14.4–21.9	12.7–20.9
Biomet (Warsaw, IN)			
ACG CR	55–75	18.1–23.9	N/A
Maxim CR	55–75	13.3–15.3	N/A
Maxim PS	55–75	15.2	N/A
Vanguard CR	55–75	13.3–15.3	13.9–20.1
Vanguard PS	55–75	16.3	10.8–17.7
Vanguard RP CR, PS	55–70	16.3	3–3.8
Kyocera Medical (Osaka, Japan)			
Bisurface PS	X small–X large	16.5	4.5–9.5
LFA CR	1–4	18–22.5	19.5–24.5
LFA PS	1–4	18	9.4–14.4

CR, cruciate retaining; PS, posterior stabilized; N/A, not available.
